# Sodium Intake and Incident Atrial Fibrillation in Individuals With Vascular Disease

**DOI:** 10.1001/jamanetworkopen.2024.21589

**Published:** 2024-07-11

**Authors:** Linda S. Johnson, Andrew Mente, Philip Joseph, David Conen, Alexander P. Benz, William F. McIntyre, Isabel Drake, Gunnar Engström, Stuart J. Connolly, Salim Yusuf, Jeffrey S. Healey

**Affiliations:** 1 Department of Clinical Sciences, Malmö, Lund University, Lund, Sweden; 2 Population Health Research Institute, McMaster University, Hamilton, Ontario, Canada; 3 Department of Cardiology, University Medical Center Mainz, Mainz, Germany; 4 Division of Cardiology, Department of Medicine, Faculty of Health Sciences, McMaster University, Hamilton, Ontario, Canada

## Abstract

**Importance:**

Numerous prospective cohort studies have reported a J-shaped association of urinary sodium excretion with cardiovascular events and mortality.

**Objective:**

To study the association between sodium intake and incident atrial fibrillation (AF).

**Design, Setting, and Participants:**

This cohort study included participants in the Ongoing Telmisartan Alone and in Combination with Ramipril Global Endpoint Trial (ONTARGET) and Telmisartan Randomised Assessment Study in ACE Intolerant Subjects With Cardiovascular Disease (TRANSCEND) multicenter, randomized clinical trials comparing the effect of ramipril 10 mg daily with telmisartan 80 mg daily, or their combination (ONTARGET) or 80 mg telmisartan daily with placebo (TRANSCEND) for the outcome of death from cardiovascular causes, myocardial infarction, stroke, or hospitalization for heart failure. ONTARGET and TRANSCEND included 31 546 participants with vascular disease or high-risk diabetes, and this study excluded participants without a urine sample for sodium measurement, missing data for key covariates, a history of AF, or AF detected in the first year after enrollment. Analyses were performed in July 2023 to May 2024.

**Exposure:**

Estimated sodium intake from a morning fasting urine sample (Kawasaki formula).

**Main Outcomes and Measures:**

The main outcome was incident AF. The association between estimated sodium intake and incident AF was modeled using multivariable adjusted Cox regression and cubic splines.

**Results:**

A total of 27 391 participants (mean [SD] age, 66.3 [7.2] years; 19 310 [70.5%] male) were included. Mean (SD) estimated sodium intake was 4.8 (1.6) g/d. During a mean (SD) follow-up of 4.6 (1.0) years, 1562 participants (5.7%) had incident AF. After multivariable adjustment, a J-shaped association between sodium intake and AF risk was observed (*P* for nonlinearity = .03). Sodium intake of 8 g/d or greater (3% of participants) was associated with incident AF (hazard ratio, 1.32; 95% CI, 1.01-1.74) compared with sodium intake of 4 to 5.99 g/d. Cubic splines showed that sodium intake greater than 6 g/d (19% of participants) was associated with a 10% increased AF risk per additional 1-g/d sodium intake (hazard ratio, 1.10; 95% CI, 1.03-1.18), but with no further lowering of AF risk at lower levels of sodium intake.

**Conclusions and Relevance:**

In this cohort study of sodium intake and AF risk, there was a J-shaped association between sodium intakes and AF risk in patients with cardiovascular disease or diabetes. Lowering sodium intake for AF prevention is best targeted at individuals who consume high sodium diets.

## Key Points

**Question:**

Is estimated sodium intake associated with atrial fibrillation (AF) risk in individuals with vascular disease?

**Findings:**

In this cohort study among 27 391 participants with vascular disease or high-risk diabetes enrolled in the Ongoing Telmisartan Alone and in Combination with Ramipril Global Endpoint Trial and Telmisartan Randomised Assessment Study in ACE Intolerant Subjects With Cardiovascular Disease trials, there was an independent J-shaped association between estimated sodium intake and atrial fibrillation incidence. Sodium intakes greater than 6 g/d were associated with 10% increase in risk for each additional 1 g of sodium consumed.

**Meaning:**

These findings suggest that lowering sodium intake for AF prevention is best targeted at individuals who consume high sodium diets exceeding 6 g per day.

## Teaser

This cohort study assesses the association of daily sodium intake with risk of incident atrial fibrillation among individuals with vascular disease.

## Introduction

Hypertension is an important potentially modifiable risk factor for atrial fibrillation (AF). Lowering blood pressure (BP) in individuals with hypertension minimizes atrial remodeling and may prevent atrial fibrillation (AF).^1^ In patients with established AF, systolic BP (SBP) is associated with stroke risk.^2^ As clinical trials have shown that reducing sodium intake can reduce BP, sodium intake is a logical target to prevent AF,^3^ stroke, and cardiac mortality.^4^

Guidelines assume a linear association between sodium intake and cardiovascular disease,^5,6,7^ and thus do not include a lower limit for recommended sodium intake. However, numerous prospective cohort studies have reported a J-shaped association between sodium intake and cardiovascular outcomes, with an increased risk of cardiovascular disease, heart failure, or death emerging at sodium intakes of less than 3 g/d and greater than 5 g/d.^8,9,10,11^ This finding has been replicated in different countries, using different methods to estimate sodium intakes, and in different study populations (eg, people with diabetes, those with vascular disease, and in the general population)^8,10,11,12,13,14,15,16,17,18,19,20,21,22^ and has also been demonstrated in meta-analyses.

The association between sodium intake and AF has not been extensively studied. We aimed to study the association between estimated sodium intake (from a fasting morning urine sample) and incident AF in a high-risk population without previous AF using data from the Ongoing Telmisartan Alone and in Combination With Ramipril Global Endpoint Trial (ONTRAGET) and Telmisartan Randomized Assessment Study in ACE Intolerant Subjects With Cardiovascular Disease (TRANSCEND) trials. We also aimed to study whether the association of AF with sodium intake was modified by SBP and prevalent hypertension.

## Methods

The trial protocols for the ONTARGET and TRANSCEND trials were approved by institutional ethics committees of each center, including approval for secondary data analyses, and all participants gave written informed consent. This study is reported following the Strengthening the Reporting of Observational Studies in Epidemiology (STROBE) reporting guideline for cohort studies.

### Study Population

The study population was derived from the ONTARGET and TRANSCEND trials, which have been described in detail previously.^23,24^ Briefly, both were randomized clinical trials that included individuals at high risk of cardiovascular events, which was defined as an age at least 55 years and either established cardiovascular disease, including coronary artery disease, peripheral artery disease, or previous stroke or transient ischemic attack, or high-risk diabetes with end-organ damage. Participants with symptomatic congestive heart failure were excluded. ONTARGET included 25 620 participants between December 1, 2001, and July 31, 2008, and compared the effect of ramipril 10 mg daily vs telmisartan 80 mg daily or their combination. TRANSCEND included 5926 individuals with angiotensin-converting enzyme (ACE) inhibitor intolerance between November 1, 2001, and May 30, 2004, and compared 80 mg telmisartan daily vs placebo. In both trials, individuals with prevalent heart failure, low ejection fraction, significant valvular disease or an elevated serum creatinine (>3.0 mg/dL; to convert to micromoles per liter, multiply by 76.25), kidney artery stenosis, proteinuria in the nephrotic range, or a BP greater than 160/100 mm Hg were excluded. We pooled data from the 2 trials since they recruited from the same sites and, excepting ACE inhibitor intolerance in TRANSCEND, used the same eligibility criteria.

**Figure 1.**
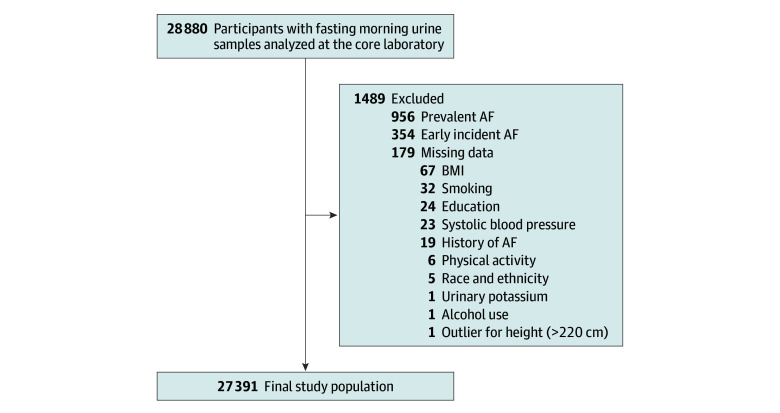
Derivation of Study Population From the Larger Ongoing Telmisartan Alone and in Combination With Ramipril Global Endpoint Trial and Telmisartan Randomised Assessment Study in ACE Intolerant Subjects With Cardiovascular Disease Trials ACE indicates angiotensin-converting enzyme; AF, atrial fibrillation; BMI, body mass index.

In 28 800 individuals (91.6%), a fasting morning urine sample was obtained before the start of the trials. We further excluded individuals with missing data for urinary potassium, ethnicity, body mass index (BMI), education, physical activity habits, smoking status, alcohol use, SBP, or history of AF, as well as outliers for height (>220 cm). We also excluded individuals with a history of AF. To test possible reverse causation, we tested our final model both with and without exclusion of individuals with an AF diagnosis within a year of study inclusion. The derivation of the study population is described in Figure 1.

### End Point Ascertainment

Follow-up in the TRANSCEND and ONTARGET trials was conducted at 6 weeks, 6 months, and every 6 months thereafter. AF was a prespecified secondary objective, and an ECG was sent to the study central office when incident AF was diagnosed during hospitalization, an emergency department visit, or in a physician’s office.^25^ AF was also detected on routine study electrocardiograms collected at each follow-up visit. The AF diagnosis has been validated in a random sample of electrocardiograms.^25^

### Data Collection

Data collection has been described in more detail previously.^15^ Fasting morning urine samples were used to estimate sodium intake using the Kawasaki formula, which has been validated against 24-hour urine collection in the Prospective Urban Rural Epidemiological Study (PURE),^26,27^ and has been shown to provide valid estimates, including in individuals using antihypertensive drugs.^28^ An international validation study reported an intraclass correlation coefficient of Kawasaki formula to actual 24-hour urine collections of 0.71.^27^ Therefore, we used the estimates derived from fasting morning urine as surrogates for sodium intake in the study. Summary details of validation of this approach are provided in the eAppendix in Supplement 1. Previous studies in this population have not shown an effect of diuretic use on the association of sodium intake with cardiovascular outcomes.^11^ All urine samples were collected prior to the trial initiation (ie, before initiation of ramipril or telmisartan) and shipped either to the Hamilton Research Laboratory or a regional laboratory in Beijing, China, using STP 250 ambient specimen shipping boxes. Sodium and potassium measurements were conducted with indirect potentiometry, using the SYNCHRON Clinical System (Beckman Coulter). Creatinine was measured with a Roche Hitachi analyzer.^15^

### Statistical Analysis

Baseline characteristics are reported across strata of estimated sodium intake as means and SDs for continuous variables and numbers and percentages for categorical variables. Two multivariable Cox frailty models, with time-to-event as time scale, were used to study the association between sodium intake and AF, based on assumptions outlined in the directed acyclic graph presented in eFigure 1 in Supplement 1.^29,30^ Model 1 adjusted for age, sex, and randomization status. Model 2 was intended to model the total association between sodium intake with AF risk (ie, including the association mediated by other risk factors) and adjusted for model 1 covariates and BMI, physical activity (moderate or strenuous physical activity habits vs sedentary), smoking (former or current smoker vs never smoker), education (9-12 years or college/trade education or more vs ≤8 years) as a marker of socioeconomic status, and alcohol use. Model 2 was our main model. Both models included a random effect for geographical region of inclusion in the ONTARGET and TRANSCEND trials. These models were applied using estimated daily sodium intake categorized into prespecified groups (<2.00, 2.00-2.99, 3.00-3.99, 4.00-5.99, 6.00-6.99, 7.00-7.99, and ≥8.00 g) with 4.00 to 5.99 g/d sodium as the reference category,^15^ as well as modeled with cubic splines, fitted using 4 prespecified knots at 2.4, 4.1, 5.2 and 7.4 g of estimated daily sodium intake, corresponding to the 5th, 35th, 65th, and 95th percentiles, respectively.^11^ Cubic spline models with 3 knots at the 10th, 50th, or 90th percentile (likelihood ratio test *P* = .48) or 5 knots at the 5th, 27.5th, 50th, 72.5th, or 90th percentiles did not result in better model fit (likelihood ratio test: 3 knots: *P* = .48; 5 knots: *P* = .48). The range of the spline curves was restricted to between 1 to 10 g of estimated daily sodium intake, roughly corresponding to the 1st to 99th percentile. To quantify the association between sodium intake and AF incidence per 1-g increase in sodium intake as a linear trend in population subsets with different levels of sodium intake, we also fitted Cox regression models with sodium intake as a continuous variable in 3 prespecified population subsets with sodium intakes of 0 to 2.99 g/d, 3.00 to 5.99 g/d and 6.00 g/d or more, based on the population distribution of sodium intake. A separate spline curve was drawn after reincluding all participants with early AF events. We also assessed the association between estimated potassium intake and AF risk, using a cubic spline with model 2 adjustment, including estimated sodium intake.

The association between sodium intake and SBP was estimated in a linear regression analysis adjusted for model 2 covariates. We then drew separate cubic spline curves for the association between sodium and incident AF after adjustment for SBP in participants without a history hypertension and those with a history of hypertension to determine whether there was an association between sodium intake that was independent of SBP.

All analyses were conducted in Stata version 17 (StataCorp), and cubic splines were drawn using the xblc plugin. Analyses were performed in 2023 to 2024. A 2-sided *P* < .05 was considered to denote statistical significance in the Cox regression models.

## Results

The final study population included 27 391 individuals (mean [SD] age, 66.3 [7.2] years; 19 310 [70.5%] male). Table 1 reports baseline characteristics across strata of estimated sodium intake, and a histogram of the population distribution of estimated sodium intake is presented in eFigure 2 in Supplement 1. The mean (SD) estimated sodium intake was 4.8 (1.6) g/d in the population overall, 4.9 (1.6) g/d among men, and 4.4 (1.6) g/d among women. During a mean (SD) follow-up time of 4.5 (1.0) years, 1562 patients (5.7%) had incident AF.

**Table 1.** Baseline Characteristics According to Strata of Estimated Sodium Intake

Characteristic	Participants by estimated sodium intake, No. (%)						
	0-1.99 g/d (n = 755)	2.00-2.99 g/d (n = 2499)	3.00-3.99 g/d (n = 5408)	4.00-5.99 g/d (n = 13 492)	6.00-6.99 g/d (n = 3200)	7.00-7.99 g/d (n = 1239)	≥8.00 g/d (n = 798)
Incident atrial fibrillation events	48 (6.4)	137 (5.5)	299 (5.5)	763 (5.7)	182 (5.7)	75 (6.1)	58 (7.3)
Age, mean (SD), y	67.3 (7.6)	67.2 (7.5)	66.7 (7.3)	66.3 (7.1)	65.8 (6.9)	65.2 (6.8)	65.3 (6.7)
Sex							
Male	347 (46.0)	1405 (56.2)	3475 (64.3)	9897 (73.4)	2539 (79.3)	1012 (81.7)	635 (79.6)
Female	408 (54.0)	1094 (43.8)	1933 (35.7)	3595 (26.6)	661 (20.7)	227 (18.3)	163 (20.4)
BMI, mean (SD)	27.3 (4.7)	27.3 (4.6)	27.5 (4.4)	28.0 (4.4)	29.0 (4.6)	29.4 (4.9)	30.2 (5.1)
Height, mean (SD), cm	162.8 (9.9)	164.6 (9.5)	166.0 (9.4)	168.1 (9.4)	169.7 (9.1)	170.5 (9.1)	170.3 (8.9)
Weight, mean (SD), kg	72.6 (15.0)	74.3 (14.8)	76.0 (14.4)	79.3 (14.5)	83.5 (15.3)	85.7 (16.3)	87.7 (16.8)
Systolic BP, mean (SD), mm Hg	138.8 (17.3)	140.3 (17.6)	141.1 (17.2)	142.0 (17.3)	143.1 (17.0)	143.1 (16.3)	143.3 (17.0)
Serum cholesterol, mean (SD), mg/dL	193.4 (54.1)	193.4 (42.5)	193.4 (42.5)	189.5 (42.5)	193.4 (42.5)	193.4 (42.5)	193.4 (42.5)
History of myocardial infarction	352 (46.6)	1161 (46.5)	2664 (49.3)	6664 (49.4)	1557 (48.7)	565 (45.6)	361 (45.2)
History of stroke or TIA	169 (22.4)	566 (22.6)	1198 (22.2)	2646 (19.6)	602 (18.8)	258 (20.8)	174 (21.8)
History of diabetes	304 (40.3)	802 (32.1)	1747 (32.3)	4869 (36.1)	1416 (44.2)	604 (48.7)	414 (51.9)
History of hypertension	594 (78.7)	1774 (71.0)	3648 (67.5)	9133 (67.7)	2336 (73.0)	941 (75.9)	657 (82.3)
Physical activity							
Moderate	161 (21.3)	579 (23.2)	1313 (24.3)	3132 (23.2)	738 (23.1)	269 (21.7)	182 (22.8)
Strenuous or severe	272 (36.0)	980 (39.2)	2291 (42.4)	6020 (44.6)	1409 (44.0)	562 (45.4)	327 (41.0)
Smoking status							
Former	313 (41.5)	1108 (44.3)	2620 (48.4)	7030 (52.1)	1679 (52.5)	690 (55.7)	419 (52.5)
Current	86 (11.4)	347 (13.9)	737 (13.6)	1617 (12.0)	366 (11.4)	135 (10.9)	81 (10.2)
Education							
College or trade school	233 (30.9)	856 (34.3)	1936 (35.8)	5139 (38.1)	1307 (40.8)	463 (37.4)	296 (37.1)
9-12 y	252 (33.4)	764 (30.6)	1669 (30.9)	4028 (29.9)	844 (26.4)	345 (27.8)	233 (29.2)
Medication use							
β-Blockers	439 (58.1)	1429 (57.2)	3177 (58.7)	7702 (57.1)	1803 (56.3)	690 (55.7)	447 (56.0)
Aspirin	570 (75.5)	1906 (76.3)	4115 (76.1)	10470 (77.6)	2443 (76.3)	960 (77.5)	599 (75.1)
Calcium channel blockers	341 (45.2)	905 (36.2)	1651 (30.5)	4333 (32.1)	1237 (38.7)	567 (45.8)	406 (50.9)
Statins	341 (45.2)	905 (36.2)	1651 (30.5)	4333 (32.1)	1237 (38.7)	567 (45.8)	406 (50.9)
Diuretics	302 (40.0)	844 (33.8)	1504 (27.8)	3395 (25.2)	857 (26.8)	377 (30.4)	338 (42.4)
Randomized treatment group							
Telmisartan	273 (36.2)	922 (36.9)	1996 (36.9)	4849 (35.9)	1168 (36.5)	481 (38.8)	284 (35.6)
Ramipril	199 (26.4)	696 (27.9)	1459 (27.0)	3677 (27.3)	860 (26.9)	354 (28.6)	204 (25.6)
Combination telmisartan and ramipril	182 (24.1)	651 (26.1)	1453 (26.9)	3708 (27.5)	875 (27.3)	306 (24.7)	216 (27.1)
Placebo	101 (13.4)	230 (9.2)	500 (9.2)	1258 (9.3)	297 (9.3)	98 (7.9)	94 (11.8)

Abbreviations: BMI, body mass index (calculated as weight in kilograms divided by height in meters squared); BP, blood pressure; TIA, transient ischemic attack.

**Figure 2.**
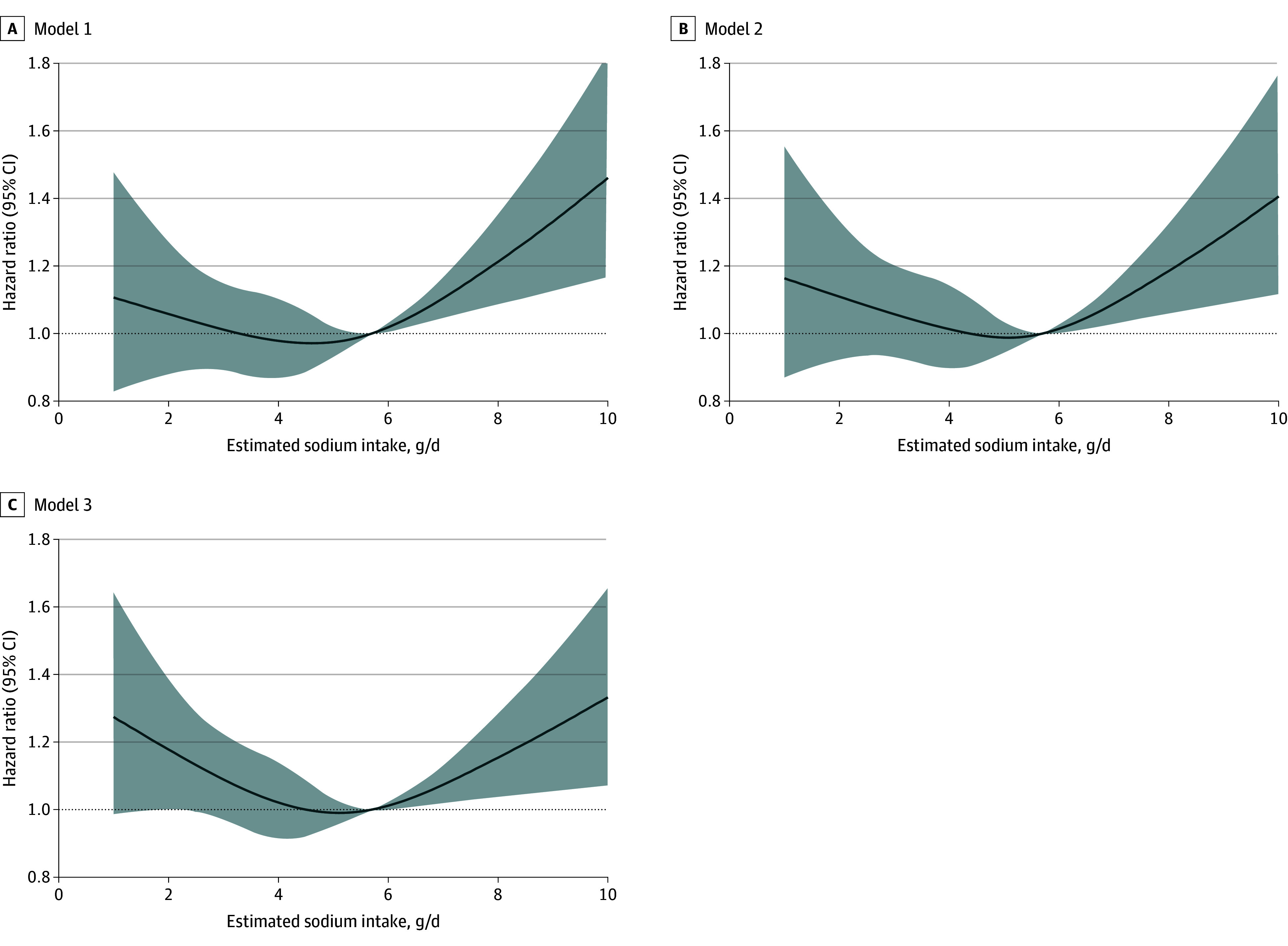
Estimated Sodium Intake and Incident Atrial Fibrillation Model 1 adjusted for age, sex, and randomization status. Model 2 adjusted for model 1 covariates and body mass index, physical activity (moderate or strenuous physical activity habits vs sedentary), smoking (former or current smoker vs never smoker), education (9-12 years or college/trade education or more vs ≤8 years) as a marker of socioeconomic status, and alcohol use. Model 3 adjusted for model 2 and included early atrial fibrillation events.

Table 2 reports results of the Cox frailty models for AF by strata of estimated sodium intake, with a sodium intake of 4.00 to 5.99 g/d as the reference category. Sodium intake of 8.00 g/d or greater (798 participants [3.X%]) was significantly associated with increased AF risk (hazard ratio [HR], 1.32 [95% CI, 1.01-1.74]) after multivariable adjustment. Sodium intake less than 2.00 g/d (755 participants [3.X%]) was not statistically significantly associated with a higher risk of AF (HR, 1.32 [95% CI, 0.98-1.77]). Restricted cubic splines showed a J-shaped association between estimated sodium intake and AF risk (*P* for nonlinearity = .03) (Figure 2). In the cubic spline analyses, estimated sodium intake of 6.00 g/d or greater (5237 participants [19.1%]) was associated with a significantly higher risk of AF, but the curve flattened out at sodium intakes less than 6.00 g/d, indicating no evidence that sodium lowering from the population mean (3.00-5.00 g/d) to levels less than 3.00 g/d was associated with reducing AF risk. Among participants with an estimated sodium intake of 6.00 g/d or greater, each 1-g increase in sodium intake was associated with 10% increased risk of AF (HR, 1.10 [95% CI, 1.03-1.18]). There was no significant association of increased sodium intake with risk of AF among 3254 participants (11.9%) with an estimated sodium intake of less than 3 g/d (HR per 1-g/d, 0.87 [95% CI, 0.66-1.13]). Most of the study population, 18 900 participants (69.0%), had an estimated sodium intake between 3.00 and 5.99 g/d, for which we found no association of increased sodium intake with AF incidence (HR per 1-g/d increase, 1.01 [95% CI, 0.94-1.10]; *P* = .70). When individuals with early AF events were reincluded, low sodium intakes became significantly associated with incident AF (HR, 1.44 [95% CI, 1.12-1.86]) (Table 2 and Figure 2C). As a subanalysis, we also tested a more extensively adjusted model, including alcohol use; history of diabetes, myocardial infarction, or stroke; SBP; use of β-blockers, calcium channel blockers, aspirin, statins, or diuretics; and model 2 covariates, using cubic splines (eFigure 3 in Supplement 1). This resulted in wider 95% CIs but a similar U-shaped curve.

**Table 2.** Cox Frailty Models for Estimated Daily Sodium Intake and Incident Atrial Fibrillation Events

Model	Estimated daily sodium intake, g													
	0-1.99		2.00-2.99		3.00-3.99		4.00-5.99		6.00-6.99		7.00-7.99		≥8.00	
	HR (95% CI)	*P* value	HR (95% CI)	*P* value	HR (95% CI)	*P* value	HR (95% CI)	*P* value	HR (95% CI)	*P* value	HR (95% CI)	*P* value	HR (95% CI)	*P* value
1^a^	1.28 (0.96-1.72)	.10	1.01 (0.84-1.21)	.93	0.99 (0.87-1.13)	.90	1 [Reference]		1.03 (0.88-1.21)	.18	1.18 (0.93-1.49)	.18	1.42 (1.09-1.86)	.01
2^b^	1.32 (0.98-1.77)	.06	1.04 (0.87-1.25)	.67	1.01 (0.88-1.16)	.88	1 [Reference]		1.00 (0.85-1.18)	.96	1.13 (0.88-1.43)	.33	1.33 (1.01-1.74)	.04
3^c^	1.44 (1.12-1.86)	.005	1.04 (0.88-1.23)	.65	1.03 (0.91-1.16)	.69	1 [Reference]		1.00 (0.86-1.15)	.95	1.14 (0.92-1.41)	.25	1.24 (0.96-1.59)	.10

^a^ Model 1 is adjusted for age, sex, and randomization group.

^b^ Model 2 is adjusted for model 1 and height, body mass index, physical activity (moderate or strenuous physical activity habits vs sedentary), smoking (former or current smoker vs never smoker), education (9-12 years or college/trade education or more vs ≤8 years), and alcohol use.

^c^ Model 3 is adjusted for model 2 and includes early atrial fibrillation events.

**Figure 3.**
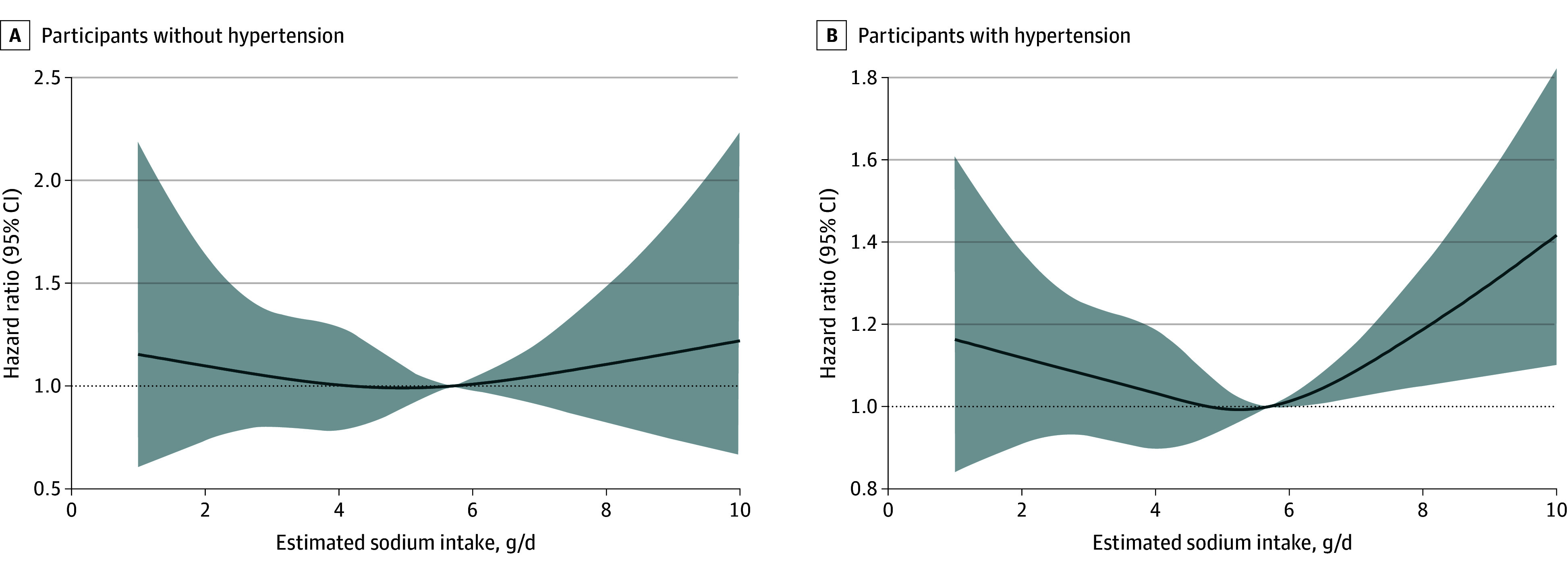
Estimated Sodium Intake and Incident Atrial Fibrillation in Participants Without and With Hypertension

Sodium intake was associated with SBP in the overall population (β = 0.70 [95% CI, 0.57-0.83] mm Hg; *P* < .001). Separate cubic spline models for incident AF were drawn in participants with and without a history of hypertension, with additional adjustment for SBP (Figure 3). Among individuals with hypertension, the association between sodium intake and AF mirrored that of the overall population, but we found no evidence of an independent association between sodium intake and AF incidence among participants without hypertension. No evidence of lower AF risk was found with low (<3.00 g/d) compared with moderate (3.00-5.00 g/d) sodium levels, irrespective of hypertension status.

Diuretics were used in 28% of participants, and the ONTARGET and TRANSCEND trial protocols did not differentiate between loop diuretics and thiazide-like drugs. There was no difference in the estimated daily sodium intake among individuals using diuretics compared with those who were not (mean [SD] sodium intake, 4.8 [1.8] g/d vs 4.8 [1.6] g/d; *P* = .71). Among diuretic users, XXX (86.X%) had a history of hypertension, and the incidence of heart failure hospitalization was higher compared with participants who did not use diuretics (1.61 [95% CI, 1.48-1.75] events per 100 person-years vs 0.53 [95% CI, 0.49-0.58] events per 100 person-years). Diuretic users without a history of hypertension had the highest incidence of heart failure hospitalization (2.46 [95% CI, 2.04-2.96] events per 100 person-years). Therefore, we consider that the subpopulation of diuretic users could be enriched not only for hypertension but also asymptomatic heart failure with slightly reduced or preserved ejection fraction. Analyses stratified on diuretic use are presented in eFigure 4 in Supplement 1. Among participants without diuretic use, we found no association of sodium intake with incident AF, while the association between high sodium intakes and incident AF was strengthened among participants with diuretic use, especially in the subgroup without a history of hypertension. Compared with moderate sodium intakes (3.00-5.00 g/d), low sodium intake (<3.00 g/d) was not associated with a lowering of AF risk, irrespective of diuretic use.

There was no evidence of an association between potassium intake and AF incidence (eFigure 5 in Supplement 1). There was no interaction between estimated sodium intake and estimated potassium intake.

Finally, to test the hypothesis that the association between estimated sodium intake and incident AF was driven by the Kawasaki model itself we tested 2 models in which the estimated sodium intake was calculated using falsified urinary sodium measurement. First, we used a falsified constant urine sodium measurement at the median urine sodium value (113 mEq/L for males and 93 mEq/L for females; to convert to millimoles per liter, multiply by 1). There was a strong correlation between the estimated sodium intakes calculated with this falsified constant and the one calculated using the actual measurements (Spearman ρ = 0.70; *P* < .001), and a similar cubic spline curve for the association between the falsified estimated sodium intakes and incident AF as that drawn using the actual urine sodium intakes for estimation of sodium intake (eFigure 6 in Supplement 1). However, considering that this method would result in using falsified urine sodium measurements that are close to the actual urine sodium measurements, we also tested a model in which the falsified sodium intake estimation was based on a randomly generated falsified urine sodium, within the range of the population distribution. This resulted in a lower degree of correlation with the estimated sodium intakes using the actual urine sodium measurements (Spearman ρ = 0.43; *P* < .001), and the cubic spline curves drawn using this falsified sodium intake did not show an association between the Kawasaki model and incident AF (eFigure 6 in Supplement 1).

## Discussion

In this cohort study among individuals with vascular disease or diabetes, we found a J-shaped association between sodium intake and AF incidence. One in 5 study participants had a daily sodium intake of at least 6.00 g, and their risk of incident AF was 10% higher with each additional gram of sodium intake above that threshold.

The Finnish OPERA study had a population of 716 individuals enriched for hypertension and assessed long-term AF risk associated with sodium intakes derived from 7-day food diary collected at a mean age of 52 years.^31^ Using this method, the top quartile of estimated sodium intake (>4134 mg per 2000 kcals/d) was associated with greater AF risk compared with the bottom 3 quartiles, in which AF risk was similar. In the UK Biobank study of approximately 470 000 individuals, the Kawasaki equation was used to estimate sodium intake from spot urine samples, and a J-shaped association between sodium intake and incident AF was reported across quintiles of estimated sodium intake.^32^ The AF incidence in the UK Biobank was low, at 21 events per 10 000 person-years in men and 11 events per 10 000 person-years in women, likely due to a low mean age (approximately 56 years at baseline, with a median follow-up time of 8 years).^32^ The findings in the UK Biobank study^32^ are thus consistent with findings in the patients with higher risk in our study, and reaffirms the J-shaped association found in this cohort. In contrast to the Finnish and UK studies,^31,32^ our study is based on a high-risk population, with a higher incidence rate of AF, which has allowed us to more reliably model the shape of the association between sodium intake and AF.

Low sodium intake, at levels consumed by less than 3% of the population in our study, was associated with increased AF risk when early AF events were included. Low sodium intakes could lead to AF through orthostatic hypotension^33,34^ and activation of the renin-angiotensin-aldosterone system, as well as increased adrenaline excretion.^35,36^ Besides observational studies that have reported J- or U-shaped associations between sodium intake and cardiovascular outcomes,^8,10,12,13,14,15,16,17,18^ there are also data from interventional studies,^37^ including a recent meta-analysis showing an increased risk of in-hospital death in patients with heart failure on sodium-restricted diets in clinical trials^38^ and data showing an inverse correlation between country-level sodium intake and life expectancy.^39^ Despite this, current guidelines recommend lowering sodium intake to a goal of between less than 1.50 g/d to less than 2.40 g/d.^6,40^ Our study found no evidence to support low sodium intakes (<3.00 g/d) compared with moderate levels (ie, at the population mean of 3.00-5.00 g/d), and there may be a higher risk of AF with low compared with moderate sodium intakes.

Randomized trials of long-term sodium lowering and cardiovascular outcomes are limited. The randomized Salt Substitute and Stroke Study found that in a high-risk population with high discretionary sodium use and low potassium intake in China, the use of a sodium-substituted salt reduced sodium excretion, SBP, and the risk of stroke and mortality.^41^ However, the findings may not be applicable to other populations with higher intakes of potassium, lower sodium, and lower use of discretionary sodium, and it is uncertain whether the observed benefit of the salt substitute in Salt Substitute and Stroke Study was due to reduced sodium intake or higher potassium intake (eg, urinary potassium change was +803 mg vs a sodium change of –350 mg).^41^ Randomized trials to specifically determine the effect of low sodium intake (ie, <2.50 g/d) compared with moderate intake on clinical outcomes are still not available, to our knowledge. Furthermore, the feasibility of such studies or of the implementation of sodium reduction in larger populations can be questioned, considering the tight neurohormonal regulation of sodium intake.^42,43^ Our findings suggest a need for trials testing effects of both high and low vs moderate sodium intakes with the use of intermediate biomarkers of AF risk and ideally long-term trials of new AF in patients at high risk. The trial could target patients with established cardiovascular disease, heart failure, or diabetes,^25,44^ as well as individuals with signs of atrial cardiomyopathy, such as supraventricular ectopy, enlarged left atria, or elevated N-terminal prohormone of brain natriuretic peptide,^45,46,47,48^ and could also be assessed using clinical risk scores, such as the Cohorts for Heart and Aging Research in Genomic Epidemiology Model for Atrial Fibrillation risk score.^49^ Reduced tissue sodium could also plausibly be achieved through the use of sodium-glucose cotransporter 2 inhibitors.^50^

Among individuals without hypertension, after adjustment for SBP, sodium intake was not associated with AF incidence, although events were limited in this subgroup of patients (389 AF events). However, the PURE^9^ and Prevention of Renal and Vascular End-Stage Disease^51^ studies similarly found that the increased risk of CVD events associated with high sodium intake (>5.00 g/d) was largely confined to participants with hypertension.

Among individuals with hypertension, the association between sodium intake and incident AF was independent of SBP. We found no effect of randomization to telmisartan or ramipril alone or in combination on AF incidence in the overall population or among participants with hypertension. The association between sodium intake and AF was also more pronounced among participants using diuretics, especially those without a history of hypertension, despite nearly identical estimated sodium intakes in participants with and without diuretic use. Since diuretic users were substantially more likely to be hospitalized for heart failure and had a higher prevalence of hypertension, we assume that these individuals had a high prevalence of unreported heart failure with preserved ejection fraction or early asymptomatic heart failure with reduced ejection fraction, and that this influenced the AF risk associated with sodium intake. Future studies are needed to address whether interventions targeting sodium could reduce AF incidence among individuals with subclinical heart failure.

### Limitations

This study has some limitations. We used a baseline measurement of sodium intake, derived from fasting morning urine samples, and the Kawasaki formula to estimate sodium intake. Urine collection before trial run in ensured that the trial medications did not influence sodium excretion. Repeated 24-hour urinary collections are considered the reference standard for estimating usual sodium intake.^48^ We deemed actual 24-hour collections to be unfeasible in a large international study of 40 countries and expected to have a greater risk selection bias due to noncompletion of 24-hour collections. For example, in a recent National Health and Nutrition Examination Survey study,^52^ completion rates for 24-hour urine samples were approximately 75%, and completers differed from noncompleters for age, sex, race and ethnicity, BMI, and hypertension status. For these reasons, the World Health Organization suggests the use of formula-derived estimates of sodium and potassium intake in population-level studies monitoring intakes over time.^53^ Our approach has been validated in an international study against actual 24-hour urine estimates of sodium and potassium excretion.^27^ The Kawasaki formula using fasting morning urine produces the least biased estimate compared with 24-hour urine collection^54^ and has been validated in previous studies of healthy individuals,^26^ individuals with hypertension,^28,54,55^ and in our international validation study.^27^ A 2013 study by Cogswell et al^56^ reported that the Kawasaki formula was associated with the largest bias compared with actual 24-hour urine collections. However, Cogswell et al^56^ used a nonfasting spot urine sample, which would be expected to produce biased overestimates since the Kawasaki formula was developed and validated for a fasting urine sample, best reflecting basal excretion. In a 2015 study^54^ that used an appropriate fasting sample, the Kawasaki formula approach was reported to be associated with the least biased estimates (vs other formulae) compared with actual 24-hour urine. Further evidence of the construct validity of our approach is the association of 24-hour urine estimates of sodium excretion to BP (2.11/0.78 mm Hg/g increase in estimated 24-hour urinary sodium excretion), which are consistent with estimates reported in randomized clinical trials of sodium reduction.^57^ Another criticism of the Kawasaki formula is that it may overestimate sodium intake compared with 24-hour urine collection, where meta-analyses of published studies report mean sodium intakes that are approximately 25% lower than in this study. In the validation study of the Kawasaki formula published by our group using the PURE cohort, the Kawasaki formula resulted in 7.6% higher estimated sodium intakes.^58^ The estimated sodium intakes in this study may therefore be somewhat overestimated, which would imply that the risk of AF increases at a lower sodium intake than we report, probably between 4.50 and 5.50 g/d. We have also found that the Kawasaki model on its own does not produce a U-shaped association with estimated sodium intake and AF^59^ by using a falsified random sodium intake in place of the factual urine sodium measurements.

Other limitations include the focus on patients with high risk of AF and existing vascular disease that may be vulnerable to the extremes of sodium intake; therefore, findings in this population may not be applicable to a generally healthy populations, which also limits our ability to estimate the total association of sodium with AF risk. Furthermore, some subclinical or asymptomatic AF events may have been missed. Conversely, there may be greater detection of AF occurring in individuals with other types of vascular events that have required hospitalization (eg, myocardial infarction and stroke).

## Conclusions

In this cohort study of sodium intake and incident AF risk, we observed a J-shaped association between sodium intake and incident AF. These data suggest that lowering sodium intake for AF prevention is best targeted at individuals who consume high-sodium diets.

## Notes

Supplement 1. **eFigure 1.** Directed Acyclic Graph Modeling the Association Between Sodium Intake and Atrial Fibrillation **eFigure 2.** Population Distribution of Estimated Sodium Intake **eFigure 3.** Estimated Potassium Intake and Incident Atrial Fibrillation **eFigure 4.** Cubic Spline Models for Incident Atrial Fibrillation in Individuals Without and With Diuretics and Individuals Without Hypertension Using Diuretics **eFigure 5.** Cubic Spline Model for Estimated Potassium Intake and AF Risk Using Model 2 Adjustment **eFigure 6.** Cubic Spline Models Drawn Using Falsified Urine Sodium Measurements, at the Sex-Specific Median and Randomly Generated **eAppendix.** The Kawasaki Method for Estimating Sodium Intake

Supplement 2.
Data Sharing Statement
