# Supplementary material for: Sodium Intake and Incident Atrial Fibrillation in Individuals With Vascular Disease
Source: JAMA Netw Open. 2024 Jul 11;7(7):e2421589. doi: 10.1001/jamanetworkopen.2024.21589 (PMC11240191; doi:10.1001/jamanetworkopen.2024.21589)
Supplement: Supplement 1. — eFigure 1. Directed Acyclic Graph Modeling the Association Between Sodium Intake and Atrial Fibrillation eFigure 2. Population Distribution of Estimated Sodium Intake eFigure 3. Estimated Potassium Intake and Incident Atrial Fibrillation eFigure 4. Cubic Spline Models for Incident Atrial Fibrillation in Individuals Without and With Diuretics and Individuals Without Hypertension Using Diuretics eFigure 5. Cubic Spline Model for Estimated Potassium Intake and AF Risk Using Model 2 Adjustment eFigure 6. Cubic Spline Models Drawn Using Falsified Urine Sodium Measurements, at the Sex-Specific Median and Randomly Generated eAppendix. The Kawasaki Method for Estimating Sodium Intake [file jamanetwopen-e2421589-s001.pdf]

Supplemental Online Content

Johnson LS, Mente A, Joseph P, et al. Sodium intake and incident atrial fibrillation in individuals with vascular disease. *JAMA Netw Open*. 2024;7(7):e2421589. doi:10.1001/jamanetworkopen.2024.21589

eFigure 1. Directed Acyclic Graph Modeling the Association Between Sodium Intake and Atrial Fibrillation
eFigure 2. Population Distribution of Estimated Sodium Intake
eFigure 3. Estimated Potassium Intake and Incident Atrial Fibrillation
eFigure 4. Cubic Spline Models for Incident Atrial Fibrillation in Individuals Without and With Diuretics and Individuals Without Hypertension Using Diuretics
eFigure 5. Cubic Spline Model for Estimated Potassium Intake and AF Risk Using Model 2 Adjustment
eFigure 6. Cubic Spline Models Drawn Using Falsified Urine Sodium Measurements, at the Sex-Specific Median and Randomly Generated
eAppendix. The Kawasaki Method for Estimating Sodium Intake

This supplemental material has been provided by the authors to give readers additional information about their work.

© 2024 Johnson LS et al. *JAMA Network Open.*

efigure 1. Directed acyclic graph for the direct association between sodium intake and atrial fibrillation

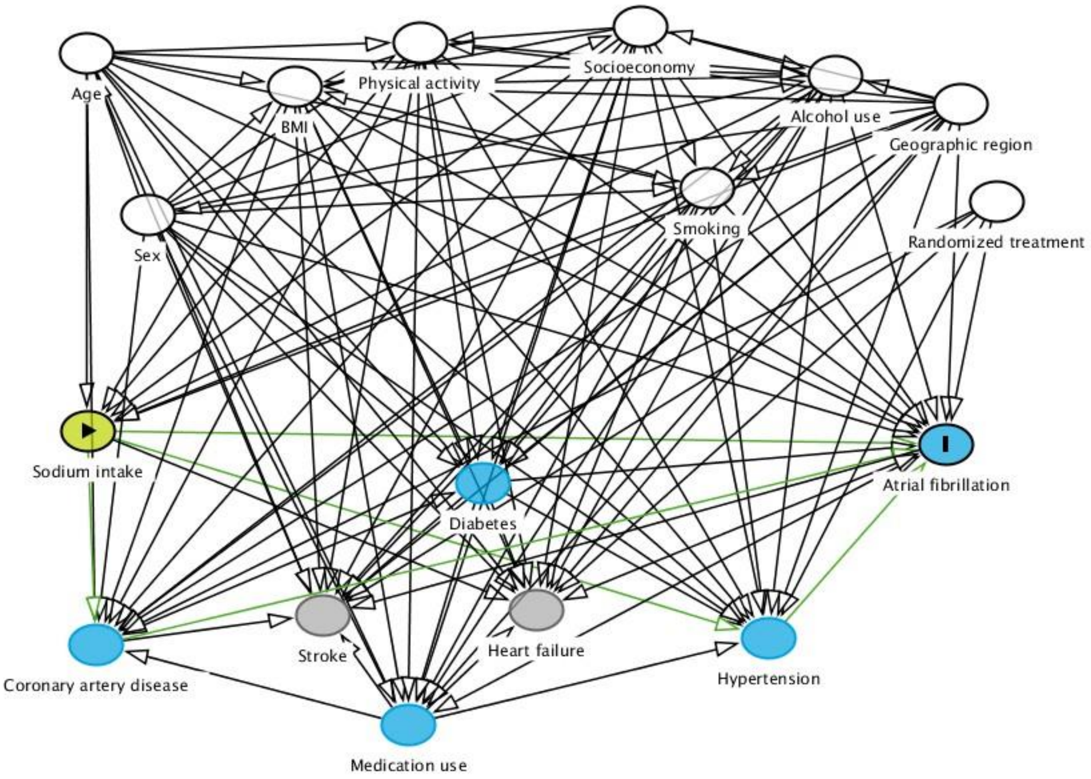

Created using dagitty.net
The green variable, sodium intake, represents the exposure, and the green arrows represent open paths between the exposure and the outcome, atrial fibrillation. Blue circled variable represent variables that need not be adjusted for in order to estimate the total effect of sodium intake on atrial fibrillation, while the variables with white circles are variables for which adjustment is necessary in order to estimate the total effect of sodium intake on atrial fibrillation.

BMI= Body mass index

© 2024 Johnson LS et al. *JAMA Network Open.*

Efigure 2 Population distribution of estimated sodium intake

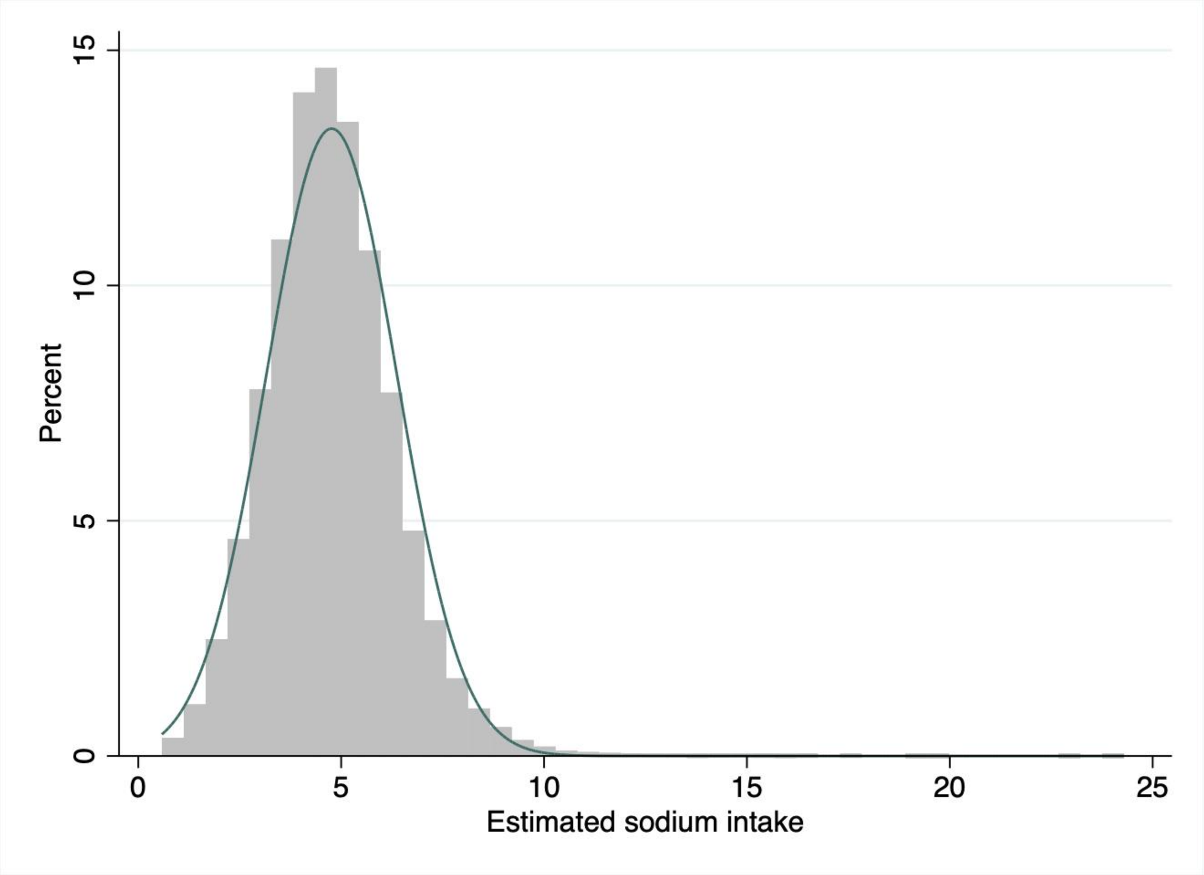

Estimated sodium intake in g/day

© 2024 Johnson LS et al. *JAMA Network Open.*

efigure 3 Cubic spline model for incident atrial fibrillation with additional covariate adjustment

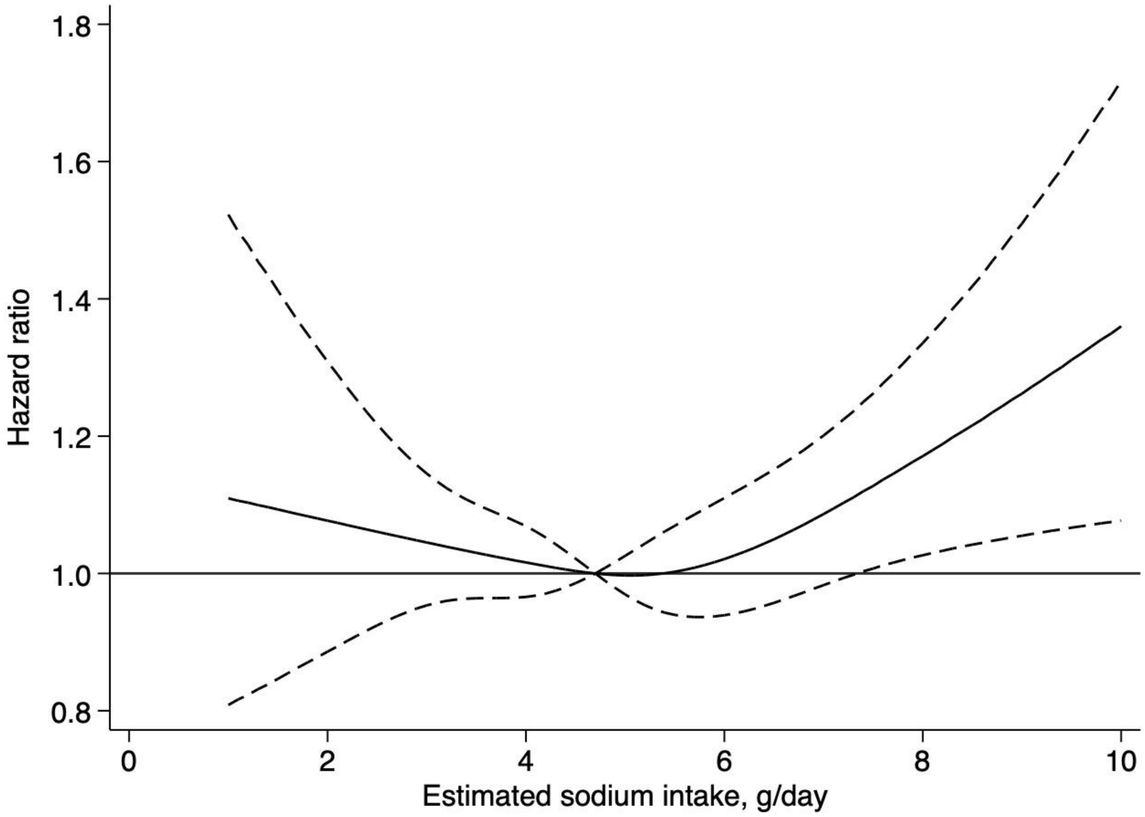

Adjusted for age, sex, randomization status in the ONTARGET/TRANSCEND trials, body mass index, systolic blood pressure, physical activity (moderate or strenuous physical activity habits vs sedentary), smoking (former or current smoker vs never smoker) and education (9-12 years or college/trade education or more vs 8 years or less), alcohol use, a history of diabetes, myocardial infarction, or stroke, systolic blood pressure, the use of beta-blockers, calcium channel blockers, aspirin, statins, and diuretics.

© 2024 Johnson LS et al. *JAMA Network Open.*

efigure 4 a-c Cubic spline models for incident atrial fibrillation in individuals without (a) and with (b) diuretics, as well as individuals without hypertension using diuretics (c)

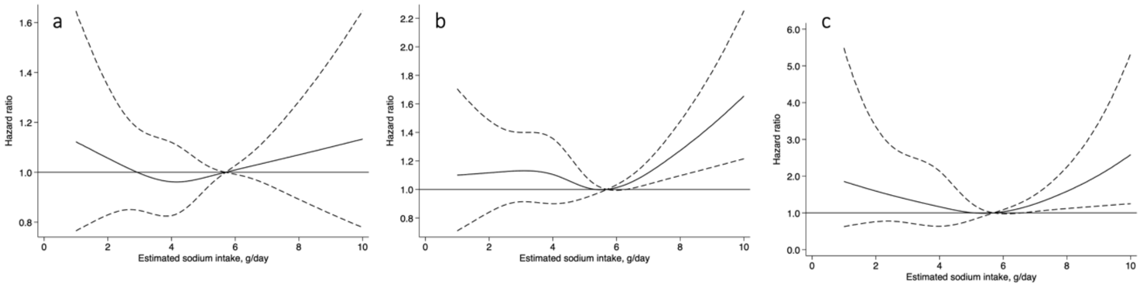

a. Includes 19,774 individuals and 1,021 events
b. Includes 7,617 individuals and 541 events
c. Includes 1,012 individuals and 66 events

Cox frailty models with a random effect on geographical region. Model 1 is adjusted for age, sex and randomization status in the ONTARGET/TRANSCEND trials. Model 2 is adjusted for Model1 + BMI, physical activity (moderate or strenuous physical activity habits vs sedentary), smoking (former or current smoker vs never smoker) and education (9-12 years or college/trade education or more vs 8 years or less) and alcohol use.

© 2024 Johnson LS et al. *JAMA Network Open.*

efigure 5. Cubic spline models for incident atrial fibrillation by estimated potassium intake

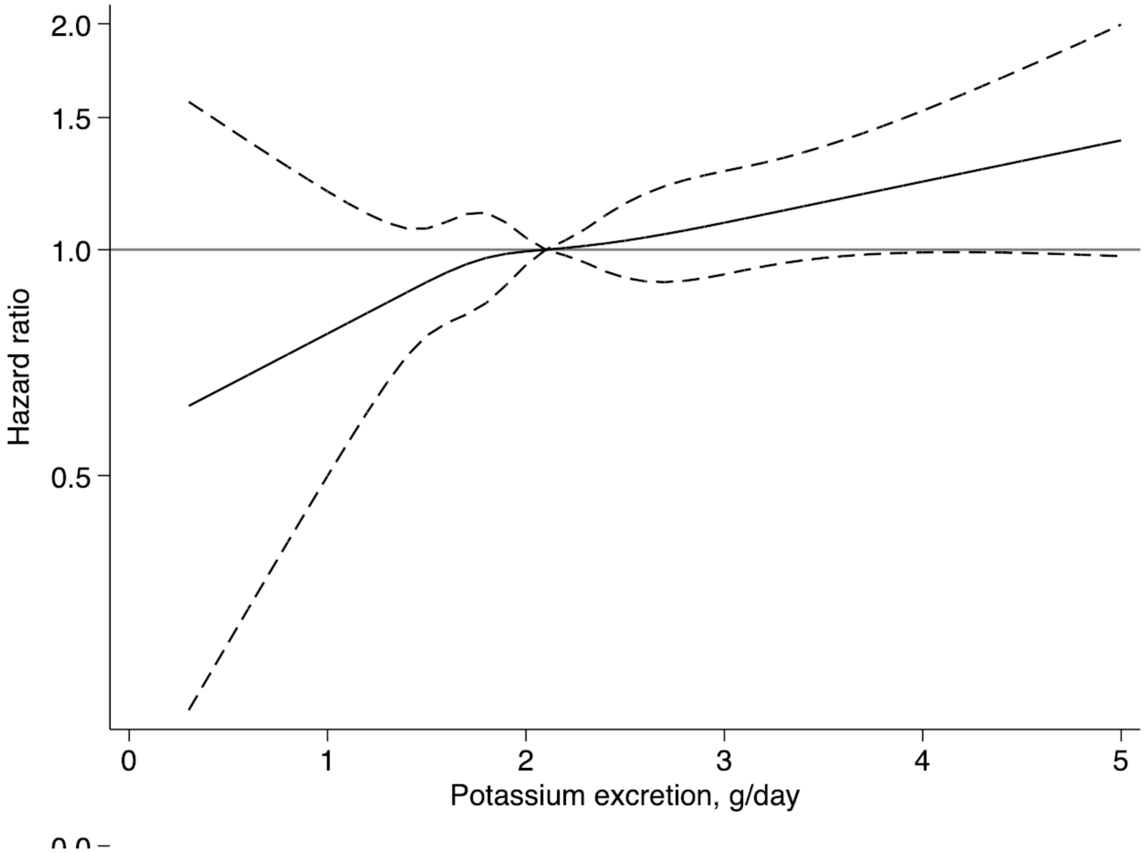

Cox frailty model with a random effect on geographical region, adjusted for age, sex and randomization status in the ONTARGET/TRANSCEND trials, BMI, physical activity (moderate or strenuous physical activity habits vs sedentary), smoking (former or current smoker vs never smoker) and education (9-12 years or college/trade education or more vs 8 years or less) and alcohol use.

© 2024 Johnson LS et al. *JAMA Network Open.*

Efigure 6 a-b. Cubic spline models drawn using falsified urine sodium measurements, at the sex-specific median (a) and randomly generated (b)

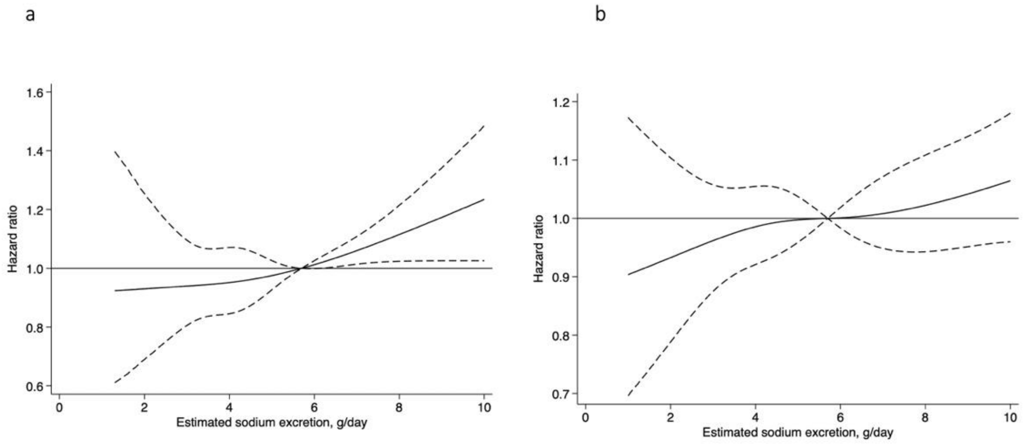

Cox frailty model with a random effect on geographical region, adjusted for age, sex and randomization status in the ONTARGET/TRANSCEND trials, BMI, physical activity (moderate or strenuous physical activity habits vs sedentary), smoking (former or current smoker vs never smoker) and education (9-12 years or college/trade education or more vs 8 years or less) and alcohol use.

© 2024 Johnson LS et al. *JAMA Network Open.*

eAppendix. The Kawasaki method for estimating sodium intake

We used the Kawasaki formula (1) to estimate24-h urinary excretion of sodium and potassium (in grams /d) from a fasting morning specimen. Previous studies (1,2) and our validation of the method in 11 countries,(3) showed that the estimated sodium excretion from the morning urine specimen shows a good correlation with direct measures of sodium excretion from the actual 24-h urine collection (intra-class correlation coefficient of 0.70;95% CI 0.61–0.77]. The BP change per g of sodium was 2.11/0.78 mm Hg,(4) which is consistent with the results of a meta-analysis of randomised controlled trials of sodium lowering in which sodium intake was measured using repeated 24 hour urine collections (5)(see summary Table below).

Summary of validity, degree of bias, and reliability results for different methods of estimated 24-hour sodium excretion versus measured excretion (From Mente A, et al, 2014. J Hypertens 32:1005-14) (3).

	24-hour measured excretion	Kawasaki method
Mean (±SD) sodium excretion, mg/day	4116± 1978	4430 ± 1253 †
Degree of bias (95% CI), mg/day	Reference	313 (182 to 444)
Validation ICC (95% CI)		
All	Reference	0.71 (0.65 to 0.76)
Excluding anti-hypertensive medication	Reference	0.73 (0.65 to 0.79)
Test-retest ICC (95% CI)		
All	0.72 (0.65 to 0.77)	0.68 (0.58 to 0.75)
Excluding anti-hypertensive medication	0.76 (0.68 to 0.81)	0.70 (0.59 to 0.77)
Pearson correlation coefficient vs. BP		

© 2024 Johnson LS et al. *JAMA Network Open.*

Systolic BP	0.14 (0.06 to 0.22)	0.16 (0.08 to 0.24)
Diastolic BP	0.18 (0.10 to 0.26)	0.19 (0.11 to 0.27)

ICC, intraclass correlation coefficient; BP, blood pressure.

† Significantly higher than 24-hour measured excretion.

‡ Significantly lower than 24-hour measured excretion and Kawasaki estimated excretion.

* Significantly greater bias than Kawasaki estimated excretion.

Measured vs. Kawasaki Sodium

Intraclass correlation coefficient (ICC) = 0.71 (95% CI: 0.65 to 0.76) (p<0.0001)

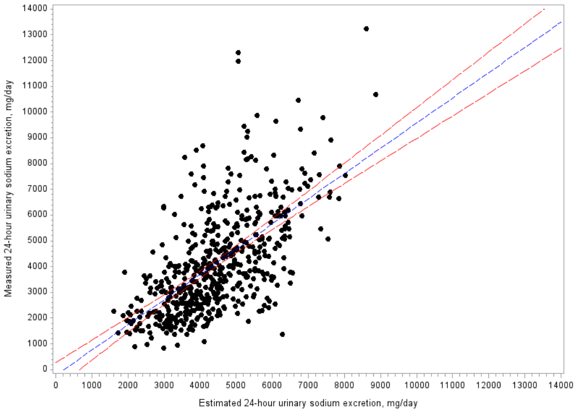

Figure. Scatter plot of estimated versus measured 24-hour urinary sodium excretion.

(From Mente A, et al, 2014. J Hypertens 32:1005-14) (3).

eReferences

1. Kawasaki T, Itoh K, Uezono K, Sasaki H. Simple method forestimating 24 h urinary sodium and potassium excretion fromsecond morning voiding urine specimen in adults.Clin Exp Pharmacol Physiol 1993; 20: 7–14.

2. Han W, Sun N, Chen Y, Wang H, Xi Y, Ma Z. Validation of the spot urine in evaluating 24-hour sodium excretion in Chinese hypertension patients. Am J Hypertens 2015; 28: 1368–75.

© 2024 Johnson LS et al. *JAMA Network Open.*

3. Mente A, O'Donnell MJ, Dagenais G, et al. Validation and comparison of three formulae to estimate sodium and potassium excretion from a single morning fasting urine compared to 24-hmeasures in 11 countries. J Hypertens 2014; 32: 1005–14.

4. Mente A, O'Donnell MJ, Rangarajan S, et al; PURE Investigators. Association of urinary sodium and potassium excretion with blood pressure. N Engl J Med 2014; 371: 601–11.

5. He FJ, Li J, Macgregor GA. Effect of longer-term modest salt reduction on blood pressure. Cochrane Database Syst Rev 2013;4: CD004937.

© 2024 Johnson LS et al. *JAMA Network Open.*
