# Supplementary material for: Sodium Intake and Incident Atrial Fibrillation in Individuals With Vascular Disease
Source: JAMA Netw Open. 2024 Jul 11;7(7):e2421589. doi: 10.1001/jamanetworkopen.2024.21589 (PMC11240191; doi:10.1001/jamanetworkopen.2024.21589)
Supplement: Supplement 2. — Data Sharing Statement [file jamanetwopen-e2421589-s002.pdf]

Data Sharing Statement

Johnson. Sodium Intake and Incident Atrial Fibrillation in Individuals With Vascular Disease. *JAMA Netw Open*. Published July 12, 2024. doi:10.1001/jamanetworkopen.2024.21589

Data
Data available: No

Additional Information
Explanation for why data not available: Data will be disclosed only upon request and approval of the proposed use of the data by a review committee.
